# PROVIDER-IDENTIFIED BARRIERS TO PALLIATIVE CARE FOR MEDICAID PATIENTS

**DOI:** 10.1093/geroni/igz038.2540

**Published:** 2019-11-08

**Authors:** Susan Enguidanos, Anna Rahman, Deborah Hoe, Kate Meyers

**Affiliations:** 1 University of Southern California, Los Angeles, California, United States; 2 California Health Care Foundation, Oakland, California, United States

## Abstract

In January 2018, California enacted Senate Bill 1004, which requires Medicaid (or Medi-Cal in California) managed care providers to offer home-based palliative care (HBPC) to their seriously ill patients. Since then, enrollment in HBPC has been lower than projected, which means many across the state continue to suffer without the pain and symptom management and psychosocial support available from a palliative care team. This study elicited clinician-perceived barriers to access to HBPC by Medi-Cal patients. We conducted a qualitative study comprising 25 individual interviews with a range of healthcare leaders and practitioners. Interviews were audio-recorded and transcribed verbatim. Using a grounded theory approach, we analyzed transcripts to determine primary themes. Our findings identified a myriad of access barriers to HBPC for the Medi-Cal population, including lack of physician knowledge about HBPC programs and benefits, a physician office structure that hampers the provision of HBPC education (i.e., one physician per office), cultural and language barriers among both physicians and patients, physicians’ lack of time, and competing demands on physicians. Providers also identified patient-related barriers, including cultural mismatch between HBPC providers and patients, trust issues related to the health-care system, and the complex challenges facing some patients such as lack of adequate and safe housing, behavioral health problems, and limited access to services that meet basic needs. These findings underscore the need for multiple approaches to increase physician education and awareness of HBPC and HBPC Medi-Cal benefits, develop culturally appropriate HBPC services, and develop programs that improve patients’ palliative care health literacy.

